# Novel Approaches Used to Examine and Control Neurogenesis in Parkinson′s Disease

**DOI:** 10.3390/ijms22179608

**Published:** 2021-09-04

**Authors:** Alla B. Salmina, Marina R. Kapkaeva, Anna S. Vetchinova, Sergey N. Illarioshkin

**Affiliations:** 1 Research Center of Neurology, 125367 Moscow, Russia; mareenqa@yandex.ru (M.R.K.); annvet@mail.ru (A.S.V.); snillario@gmail.com (S.N.I.); 2 Research Institute of Molecular Medicine & Pathobiochemistry, Prof. V.F. Voino-Yasenetsky Krasnoyarsk State Medical University, 660022 Krasnoyarsk, Russia

**Keywords:** Parkinson′s disease, α-synuclein, neurogenesis, neural stem cell, neural progenitor cell, astrocyte, optogenetics

## Abstract

Neurogenesis is a key mechanism of brain development and plasticity, which is impaired in chronic neurodegeneration, including Parkinson’s disease. The accumulation of aberrant α-synuclein is one of the features of PD. Being secreted, this protein produces a prominent neurotoxic effect, alters synaptic plasticity, deregulates intercellular communication, and supports the development of neuroinflammation, thereby providing propagation of pathological events leading to the establishment of a PD-specific phenotype. Multidirectional and ambiguous effects of α-synuclein on adult neurogenesis suggest that impaired neurogenesis should be considered as a target for the prevention of cell loss and restoration of neurological functions. Thus, stimulation of endogenous neurogenesis or cell-replacement therapy with stem cell-derived differentiated neurons raises new hopes for the development of effective and safe technologies for treating PD neurodegeneration. Given the rapid development of optogenetics, it is not surprising that this method has already been repeatedly tested in manipulating neurogenesis in vivo and in vitro via targeting stem or progenitor cells. However, niche astrocytes could also serve as promising candidates for controlling neuronal differentiation and improving the functional integration of newly formed neurons within the brain tissue. In this review, we mainly focus on current approaches to assess neurogenesis and prospects in the application of optogenetic protocols to restore the neurogenesis in Parkinson’s disease.

## 1. Introduction: Neurogenesis in the Healthy and Parkinson’s Disease-Affected Brains

### 1.1. Key Characteristics of Adult Neurogenesis

Neurogenesis is a mechanism of brain development and plasticity. Embryonic neurogenesis provides new neurons for brain growth, whereas adult neurogenesis is required for memory consolidation and tissue repair, mood regulation, and social recognition [1,2,3,4]. Functional competence of adult-born neurons results in their successful integration into pre-existing neural circuits, for instance, in the hippocampus, which is associated with activity-mediated acceleration of dendritic spines formation [5]. Therefore, the efficacy of neurogenesis in the embryonic brain corresponds to appropriate brain development and maturation. Adult neurogenesis could be considered as efficient if brain plasticity meets the current needs in cognition, social interactions, expression of emotions, memory consolidation and retrieval, forgetting, and experience-driven circuits remodeling.

**Figure 1:**
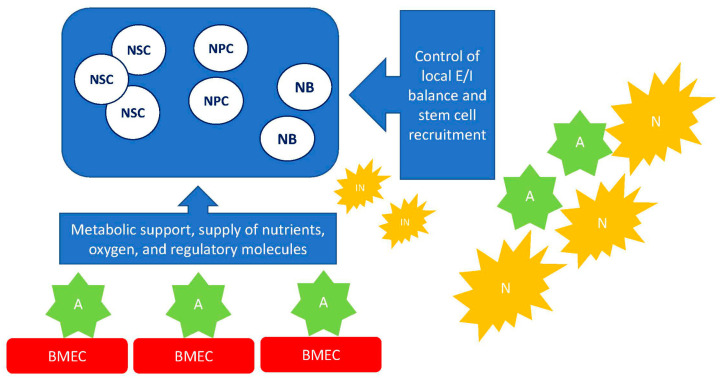
A simplified scheme of intercellular communications within the neurogenic niche. The local microenvironment is established due to the activity of neuronal, astroglial, and endothelial cells that are able to release various molecules (growth factors, neurotransmitters, cytokines, gliotransmitters, metabolites) affecting cell fate within the niche. Abbreviations used: NSC—neural stem cell, NPC—neural progenitor cell, NB—neuroblast, IN—immature neuron, N—neuron, A—astrocyte, BMEC—brain microvessel endothelial cell, E/I—excitation-inhibition balance.

The key pool of cells that could be activated to provide new neurons and astrocytes is represented by neural stem cells (NSCs) that are found in highly specialized neurogenic niches (subventricular zone, SVZ, and subgranular zone of the hippocampus, SGZ), as well in some other brain regions (cerebellum, substantia nigra, cortex), and in loci with the facilitated access to regulatory molecules, i.e., in the periventricular area with ischemia- or neuroinflammation-mediated compromised blood-brain barrier (BBB) [6,7]. NSCs exhibit two pivotal characteristics: (1) the ability to self-renew and to produce copies of themselves by symmetric or asymmetric division; (2) multipotency to produce neural progenitor cells (NPCs) that are able to differentiate into neurons, astrocytes, or oligodendroglia [8,9,10]. The proliferation of NSCs is under the tight control of the local microenvironment consisting of numerous soluble molecules: neurotransmitters (GABA, glutamate, dopamine, etc.), neuropeptides (oxytocin, angiotensin, etc.), cytokines (interleukins, chemokines, etc.), metabolites (lactate, NAD^+^), extracellular matrix proteins, and accessory cells (astrocytes, brain microvessel endothelial cells) aimed to prevent non-reasonable utilization of the NSCs pool, to coordinate cell fate, and to drive cell proliferation, differentiation, and migration on demand (Figure 1). In addition to the above-mentioned factors, local hypoxia in the neurogenic niche serves as a signal to control the NSC’s recruitment [11].

Postnatal ontogenesis, neurodegeneration, and aging are associated with progressive loss of NSCs (radial glia cells, RGCs) in the rodent hippocampus [10], thereby suggesting that mechanisms preventing the depletion of the NSCs pool came to be less efficient in older brains. Indeed, in human SVZ, the density of RGCs reduces from mid-gestation until the perinatal period, and in the human SGZ, the decline of RGCs number is observed from early ages of development until 5 years old and then in adulthood [12].

The predominant view on neurogenic events in the adult brain states that enhanced neurogenesis is required for (re)cognition and memory, whereas reduced neurogenesis manifests aberrant brain plasticity [13]. However, recent data suggest that the general picture is not so simple, at least in some details. Firstly, even the addition of new neurons to the dentate gyrus of the hippocampus in vivo provides a fresh substrate for new memories, blocking adult neurogenesis in rats results in the elongation of long-term potentiation (LTP); therefore, newly-formed cells are required for the phenomenon of hippocampal clearance and consolidation of memory in extra-hippocampal brain regions [14]. It links elevated hippocampal neurogenesis to mechanisms of forgetting when newly-arrived young cells make a vacant position for new memories by eliminating recently-learned information, but not remotely acquired ones, which already exist in extra-hippocampal brain structures [15]. Secondly, adult-born neurons inhibit the dentate gyrus activity by recruiting local interneurons, and it seems to be important for preventing memory interference and engrams overlapping in subsequent learning episodes (so-called cognitive flexibility) [13]. This mechanism underlies the ability of young dentate gyrus cells to support pattern separation and the ability of old dentate gyrus cells to support rapid recall and pattern completion [16]. That is why hyperexcitable dentate gyrus results in cognitive deficits and the impairment of pattern separation in mice [17].

### 1.2. Aberrant Neurogenesis in Parkinson′s Disease

Parkinson’s disease (PD) is a chronic neurodegenerative disease primarily affecting dopaminergic neurons in the substantia nigra pars compacta (SN) and leading to prominent motor and cognitive dysfunction. Several hypotheses have been proposed to explain the progressive cell loss in SN, including mitochondrial alterations, genetic predisposition, accumulation of abnormal proteins, development of oxidative stress, chronic neuroinflammation, and aberrant neurogenesis [18,19,20]. PD belongs to the group of neurodegenerative diseases with Lewy body and Lewy neurite pathology that are associated with the accumulation of wild-type α-synuclein protein as intracellular neuronal and glial filamentous deposits (other examples are dementia with Lewy bodies, multiple system atrophy) [21].

Presynaptic protein α-synuclein encoded by the SNCA gene belongs to the group of so-called “natively unfolded proteins” that lack ordered structure, has high flexibility and the ability to get the conformation upon binding to ligands, and are prone to aggregation and deposition [22]. In physiological conditions, it may have two states: the unfolded state in the cytosol or the helical multimeric state at the cell membranes due to its interactions with lipid rafts and phospholipids [23]. It is interesting that binding to membranes with a larger diameter (~100 nm) produces an elongated helix conformation in α-synuclein, whereas binding to membranes with small and highly curved vesicles (i.e., synaptic vesicles) results in a broken helix conformation [23]. Being located in close vicinity to vesicles in the presynaptic terminal, α-synuclein significantly affects synaptic transmission via the regulation of vesicle formation and neurotransmitter release [24]. Neural activity triggers the dispersion of α-synuclein from synapses during exocytosis in a Ca^2+^ entry-dependent manner [24]. In PD, a mutated form of α-synuclein has a tendency to aggregate and not disperse from synaptic boutons, thereby leading to deposition of synuclein-containing protofibrils [25].

In familial autosomal dominant PD, several missense mutations and multiplications of SNCA have been reported even though they are a rare cause of the disease, but exonic duplication and deletion mutations in parkin (PRKN), protein deglycase (DJ-1), and PINK1 genes have been identified in the early-onset parkinsonism [26]. SNCA multiplications are also present in some cases of sporadic PD [27].

Tissue accumulation of defective α-synuclein is one of the key features of PD. Being present intracellularly or in the extracellular space, this protein produces prominent neurotoxic effects, alters synaptic plasticity, affects autophagy, induces mitochondrial dysfunction and endoplasmic reticulum stress, deregulates intercellular communication, and supports the development of neuroinflammation, thereby providing propagation of pathological events leading to the establishment of a PD-specific phenotype [21,26,28,29]. Moreover, there is a documented transneuronal propagation of abnormal α-synuclein aggregates in PD, leading to prion-like synuclein dissemination within the nervous tissue [30,31]. The distribution of α-synuclein in the tissue might depend on the connexin 32 (Cx32)-based activity of gap junctions between cells and within the astroglial syncytium [32]. As we and others have shown before, this is confirmed by extra-brain localization of α-synuclein in PD patients, thereby suggesting the retrograde dissemination of α-synuclein forms olfactory bulbs and intestinal autonomic neurons on the brainstem structures and determining the staging of synucleinopathy development [30,33,34,35].

The role of α-synuclein in the regulation of adult neurogenesis has been partly identified: (i) SGZ neurogenesis is increased in α-synuclein knock-out mice, whereas overexpression of wild type synuclein results in decreased dendritic growth [21]; (ii) injections of α-synuclein oligomers in mice produces a significant increase in the number of proliferating cells and immature neurons in the SGZ, corresponding to the loss of dopaminergic neurons in SN [36]; (iii) expression of wild type and mutant α-synuclein in embryonic stem cells results in their reduced proliferation and neuronal differentiation associated with lowered Notch signaling in vitro [37]; (iv) in mice overexpressing wild type α-synuclein, the number of transcription factor paired box protein (Pax6)-expressing NPCs in SGZ increases, presumably, due to the deregulation of dopamine levels or altered excitation/inhibition balance in the hippocampus [38]; (v) in the experimental (MPTP) model of PD in mice, SVZ NPCs show a reduced capacity of proliferation in aged but not young animals, whereas in transgenic mice overexpressing mutant (A53T) α-synuclein and treated with MPTP, neurogenesis is reduced in olfactory bulbs and SN [39]; (vi) defects in neurogenesis seen in the olfactory bulbs and hippocampus of transgenic mice with the overexpression of wild type of α-synuclein have been attributed to the development of olfactory deficits in PD [21].

**Figure 2:**
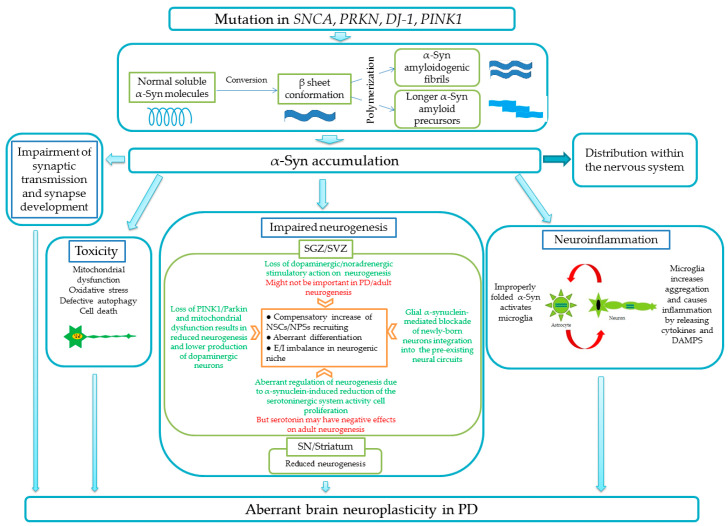
Impairment of brain plasticity in PD caused by the accumulation of α-synuclein in the brain tissue. Aberrant neurogenesis in SGZ/SVZ as well as in the substantia nigra /striatum is a result of numerous mechanisms triggered by improperly folded α-synuclein (α-syn) that are associated with neuroinflammation, direct cell toxicity, and synaptic dysfunction. Abbreviations used: *SNCA—synuclein* gene, *PRKN—parkin* gene, *DJ-1—deglycase* gene, PINK1—PTEN-induced kinase *1* gene, E/I—excitation/inhibition balance, DAMPs—damage-associated molecular patterns, SN—substantia nigra.

Figure 2 illustrates the current understandings of the role of α-synuclein in brain (patho)physiology.

Neurogenesis in a PD-affected brain is believed to be altered by several mechanisms that are not fully understood and are even based on controversial experimental findings: (i) loss of dopaminergic and noradrenergic stimulatory action on SGZ neurogenesis [40] and SVZ neurogenesis [41], however, some studies suggest that this mechanism might not be important in PD and in the adult neurogenesis, in general [42,43], or dopaminergic neurodegeneration increases SVZ- and midbrain-derived progenitor cell proliferation [44]; (ii) aberrant regulation of neurogenesis in neurogenic niches due to the α-synuclein-induced reduction of the local serotoninergic system activity, which is required for SGZ cells proliferation [45,46], however, there are some data on the negative effect of serotonin on adult neurogenesis [47]; (iii) loss of PTEN-induced kinase 1 (PINK1) and parkin, as well as mitochondrial dysfunction results in reduced SGZ and SVZ neurogenesis or suppressed production of dopaminergic neurons [48,49]; (iv) glial α-synuclein-mediated blockade of newly-born neurons integration into the pre-existing neural circuits [50].

While analyzing all these data, including the controversial data, one should keep in mind that: (i) α-synuclein demonstrates physiological activity towards newly-formed neurons, and promotes dendrite and spine development and maturation depending on the expression level [51], therefore, impairment of neurogenesis could be caused by the action of supraphysiological concentrations or aggregates of this protein, which is specific for PD pathogenesis [21]; (ii) analysis of cell proliferation and NSCs/NPCs number might not be relevant in the assessment of neurogenesis efficacy, since preserved neurogenesis could be linked to predominant self-renewal and prevention of excessive recruitment of stem and progenitor cells; (iii) compensatory increase in striatal neurogenesis and intensive migration of SVZ-generated neuroblasts to SN might be evident at the initial stages of development of the neurodegenerative process as it was shown in 1-methyl-4-phenyl-1,2,3,6-tetrahydropyridine (MPTP)-induced and 6-hydroxydopamine (6-OHDA)-induced mouse models of PD, or in human brain samples [21,52,53,54,55]. Such a compensatory increase in neurogenesis seems to utilize Wnt1-dependent and growth factors-triggered signaling machinery [40]. The contribution of other neurogenic regions of the brain (i.e., periventricular parts of the aqueduct and the fourth ventricle) into the compensatory increase in neurogenesis is not evident in experimental PD [56].

In the α-synuclein transgenic rat PD model, the impairment of SGZ neurogenesis due to excessive cell loss is evident prior to the development of motor symptoms being associated with the serotonergic deficit in the hippocampus and anxiety-like phenotype [57]. It is interesting to note that SVZ neurogenic niches are under the control of dopaminergic neurons located in the substantia nigra [58]. Thus, one could speculate that altered neurogenesis at the early (pre-motor) stage of PD would result in the insufficient production of dopaminergic neurons, but later, when the number of these cells comes to be very low, the loss of dopaminergic stimulation of SVZ niche activity would lead to the secondary suppression of neurogenesis. In sum, the widely-accepted view on the neurogenesis alterations in PD states that survival, recruitment, and proliferation of NSCs/NPCs is greatly affected by the accumulation of improperly folded proteins or signaling pathways associated with neurodegeneration and neuroinflammation, thereby leading to abnormal brain plasticity and motor and cognitive impairments [21,59].

In addition to the numerous experimental data obtained in rodent PD models, aberrant neurogenesis was found in the brain of patients with PD. Particularly, in humans, the number of the RNA-binding protein Musashi-immunopositive cells (NSCs/NPCs) within the SVZ positively correlates with the extent of dopaminergic treatment, whereas disease duration shows a negative correlation; the number of the transcription factor Sox2-immunopositive cells (NSCs) in the SGZ is significantly decreased compared with a control group [59]. Since Sox2 inhibits paracrine and autocrine Wingless/Int-1 (Wnt) signaling and maintains the cells in the proliferative state [60], one may suggest that the recruitment of stem and progenitor cells in PD is diminished.

Another important mechanism of neurogenesis impairment is directly linked to the pathology of SN as a non-conventional neurogenic niche in the adult brain: the generation of dopaminergic neurons has been shown locally in the SN by means of tracing analysis revealing newly-generated neurons either in a normal or degenerated brain in PD [61]. The physiological rate of neurogenesis in SN is several orders of magnitude lower than in SGZ but contributes to the replacement of dopaminergic neurons during the lifespan in mice [62]; however, some other studies show no evidence for local neurogenesis in the SN [63]. Neurogenesis in the SN depends on the presence of angiogenic factors and the establishment of new microvessels, thereby resembling the situation in SVZ and SGZ [64]. Recent findings on dopamine-stimulated hippocampal SGZ and striatal neurogenesis [65] suggest that PD-associated impairment of neurogenesis might have links to insufficiency of dopaminergic mechanisms of neurogenesis regulation. Stimulation of dopaminergic receptors results in the induction of neurogenesis within the SN in rodents [66]; however, it was proposed that the local microenvironment in the midbrain supports gliogenesis, but not neurogenesis [40,67]. Data on the experimental transplantation of adult rat-derived NPCs into the lesioned striatum demonstrate that special solutions should be found to drive the differentiation of grafted cells toward the desired phenotype (neuronal but not glial) [51].

## 2. iPSC-Based Platform for Studying PD-Affected Neurogenesis In Vitro

### 2.1. Generation of iPSC-Derived Dopaminergic Neurons

The development of protocols for induced pluripotent stem cells (iPSCs) generation had revolutionized the methodology of studying the brain. Particularly, the generation of neurons and glial cells from a patient became possible, thereby allowing the reconstruction of key processes of brain plasticity, development of brain tissue in vitro models, and establishment of isogenic platforms for cell-replacement therapy and cell transplantation [68]. As we have shown before, such an approach was effective in the generation of PD-derived iPSC lines with different mutations for studying defects in neurotrophic factors signaling affecting neuronal development [19], personalized modeling of PD pathogenesis [69], and screening of drug candidates [70,71]. The optimized protocols for getting dopaminergic differentiated neurons from iPSCs have been suggested [72,73], and they include the recruitment of stem cells with the transforming growth factor-beta (TGFβ) antagonists, activation of Hedgehog, Wnt, and fibroblast growth factor 8 (FGF8) signaling pathways or expression of Lmx1a, Foxa2, and Nurr1 and other midbrain-specific transcription factors for getting the midbrain floor-plate progenitors, followed by the application of neurotrophic factors (brain-derived neurotrophic factor BDNF, glia cell line-derived neurotrophic factor GDNF) and Notch receptor antagonists to induce the terminal differentiation of cells toward a dopaminergic phenotype. However, the final populations of cells are rather heterogeneous, consisting of post-mitotic neurons of different subtypes and immature cells; therefore, 3D cultures, including cerebral organoids, have been applied to improve the quality of the final cellular composition [72].

After the differentiation in vitro, dopaminergic neurons appeared to be not fully matured; therefore, acute progerin overexpression or co-culture with isogenic astrocytes is highly recommended [73,74,75]. There is growing evidence that co-culturing with astrocytes results in the promotion of neuronal differentiation and functional maturation of newly-formed iPSC-derived neurons in various models [75,76,77,78]. Thus, various local factors and types of intercellular communication affect the development of iPSC-derived dopaminergic neurons [79].

The overexpression of α-synuclein could be achieved in iPSC-derived neurons with SNCA multiplication, i.e., triplication of the gene leads to abnormally high expression and deposition of α-synuclein in differentiated cells [80], such as in PARK4 PD patients [81], hereby providing a relevant model of PD pathogenesis. In addition, severe changes in neuronal differentiation and maturation have been detected upon SNCA triplication, whereas the knock-down of SNCA mRNA in iPSC-derived cells prevents such abnormalities [82]. A53T point mutation in the SNCA gene in iPSC-derived neurons results in the development of pathological alterations in cell metabolism and defective proteostasis, early neurite degeneration, and down-regulation of some synaptic proteins [28]. Interneuronal spreading of α-synuclein within and between iPSCs cortical neurons was reproduced in the in vitro microfluidic systems allowing unidirectional axonal growth [83].

### 2.2. Generation of iPSC-Derived Midbrain Astrocytes

The establishment of a co-culture of midbrain neurons and astrocytes of the same origin would have several advantages in developing the platform for PD study and drug testing in vitro [73].

The differentiation of astrocytes from human iPSC-derived progenitors has been demonstrated in [84,85,86]. Basically, the protocols utilize the application of a medium with low fetal bovine serum (FBS) (1%–2%) and the replacement of half of the conditioned medium with the fresh one to induced astroglial phenotype acquisition within 1 month in vitro. iPSC-derived astrocytes express astroglial markers (S100 calcium-binding protein B (S100β), gap junction protein connexin 43, and water channel aquaporin 4 (AQ4)) and other molecules whose pattern resembles quiescent astrocytes. Being functionally competent, these cells respond to inflammatory stimuli by cytokine release and demonstrate Ca^2+^ elevations in basal conditions or after the stimulation with ATP or glutamate.

Another compatible approach is based on embryonic stem cells converted into neural progenitors through the stage of embryoid body (EB) formation and adding transforming growth factor (TGF) and bone morphogenetic protein 4 (BMP4) inhibitors SB431542 and LDN193189 to suppress the production of cystic embryonic bodies, followed by differentiation supported by epidermal growth factor (EGF) and ciliary neurotrophic factor (CNTF). The astrocytes obtained express astroglial markers and appropriately responded to pro-inflammatory stimuli [87]. Direct conversion of embryonic and postnatal mouse and human fibroblasts into astrocytes in vitro was proposed in [88] with NFIA, NFIB, and SOX9 transcription factors. The astrocytes obtained in this protocol demonstrate gene expression pattern, K^+^ and Ca^2+^ membrane permeability, glutamate transport, and response to cytokines stimulation similar to native brain astrocytes.

The generation of midbrain astrocytes from human iPSCs was demonstrated from small molecule-treated NPCs (smNPCs) that are able to differentiate by the withdrawal of the small molecules used for their expansion (TGF and BMP inhibitors SB431542 and dorsomorphin, Wnt stimulator and glycogen synthase kinase 3 (GSK3) inhibitor CHIR 99021, SHH stimulator purmorphamine) into midbrain dopaminergic neurons, midbrain astrocytes (that could be obtained in a medium with 4% fetal calf serum (FCS) and CNTF later replaced with dibutyryl cyclic AMP), and oligodendrocytes [89].

In another protocol, midbrain astrocytes have been obtained from SNCA-mutated iPSCs generated from PD patient’s fibroblasts according to the following conditions: fibroblast growth factor 8 (FGF8) for getting the midbrain identity, epidermal growth factor (EGF), leukemia inhibitory factor (LIF), FGF2 + heparin for effective gliogenesis, and histone deacetylase (HDAC) inhibitor valproic acid for increased expression of glial cell line-derived neurotrophic factor (GDNF). The obtained cells express astrocyte markers, such as aldehyde dehydrogenase 1 family member L1 (ALDH1L1), Vimentin, Connexin 43 (Cx43), and aquaporin 4 (AQP4), as well as S100β, accumulate α-synuclein, and release the excess of Ca^2+^ into cytosol, but demonstrate pathological mitochondrial fragmentation and aberrant respiration [90].

PD patient-specific astrocytes derived from iPSCs with mutations in the LRKK2 gene and further run in the neuron-astrocyte co-culture system support the development of a neurodegeneration characteristic for PD (incl. morphological alterations, α-synuclein accumulation, shortened neurites, and reduced cell survival), whereas astrocytes themselves demonstrate signs of incomplete autophagy [91].

In summary, the establishment of differentiated astrocytes from PD patient-derived iPSCs allows studying the astroglial contribution to the local control of neurogenesis, promoting differentiation of dopaminergic neurons co-cultured with astroglia, and developing novel methodological approaches to modulate astroglial activity with optogenetic protocols (as discussed below).

### 2.3. Generation of iPSC-Derived Midbrain Cerebral Organoids

Cerebral organoids represent another type of brain tissue model with reconstituted processes of neurogenesis and brain development. Actually, cerebral organoids reproduce embryonic neurogenesis, and the data obtained cannot be extrapolated directly to mechanisms of adult neurogenesis [92]. The key characteristics of cerebral organoids are the ability of stem cells to produce self-organized structures resembling various brain regions. This methodology is based on the production of embryonic bodies and clusters of neuroepithelial cells followed by the establishment of apico-basally polarized neural tissue that is achieved by so-called un-guided or guided protocols to get spontaneous differentiation or specification of cell development, respectively [93]. The main advantage in using human iPSC-derived cerebral organoids is an opportunity to get the human-specific cell types and tissue developmental traits that could not be reproduced in the rodent tissue [94].

In the protocols of organoids generation, the application of growth factors and morphogens is rather limited because of a shortage of knowledge on their action in a stage-specific manner and general disorganization of cells positioning with the organoids. However, successful attempts to produce cortical organoids, hippocampal organoids, ventricular zone-like regions, and their assembloids resembling some periods of brain development with the specific transcriptomic and proteomic changes have been reported and analyzed [93,94,95,96].

Another methodological problem with the lack of microvasculature and microglia in neuroectodermal progenitors-derived organoids is currently getting some solution with the approaches to prevent organoid core hypoxia by co-culturing with brain microvessel endothelial cells [97,98,99], or to support normal neuronal development with microglia cells incorporated into organoids in vitro [100]. Moreover, data on the presence of ectodermal, mesodermal, and endodermal progenitors at the earliest stages of organoids development [101] suggest that mesodermal progenitors might be able to develop into microglial cells simultaneously with neurons and astrocytes or into BMECs to provide a vascular scaffold for developing and maturing cells.

Cerebral organoids contain various cells (radial glia, intermediate progenitors) whose self-organization results in establishing the structures resembling brain development during the first trimester of human gestation [93]; therefore, they are mainly applied in studying the molecular pathogenesis of neurodevelopmental disorders. However, neurodegeneration associated with impaired neurogenesis might be examined with the cerebral organoid methodology; even the trajectory of brain cells development and their diversity are quite different in the embryonic and adult brain.

Actually, it is hard to imagine that generation of region-specific cerebral organoids from patient-derived iPSCs would give the same phenotype of neuronal and glial cells that are seen in advanced neurodegeneration. However, it was confirmed that this in vitro model provides unique opportunities for analyzing the entire mechanisms of brain plasticity under the conditions of abnormal expression of genes and proteins in the particular type of neurodegeneration. For instance, organoids generated from Alzheimer’s disease patients and aged in culture (up to 60–90 days) produce significantly higher levels of beta-amyloid and show sensitivity to inhibitors of gamma-secretase [102]. Cerebral organoids obtained from iPSCs from patients with frontotemporal dementia allow novel aspects of tau-mediated pathology to be revealed [103]. At present, the establishment of organoids correctly resembling aging- or neurodegeneration-associated changes in cell development is a great challenge for the current neurobiology and bioengineering, as was discussed recently in [104].

In modeling Parkinson’s disease with cerebral midbrain organoids, the specification of cells induces the expression of transcription factors FOXA1/2, LMX1A, and LMX1B in midbrain dopaminergic progenitor cells that are able to express tyrosine hydroxylase and produce dopamine [105]. Numerous attempts have been applied to increase the yield of tyrosine hydroxylase-immunopositive cells in organoids and to produce earlier differentiated midbrain organoids in vitro [106,107]. In some cases, cerebral organoids have been used for in vitro modeling of Parkinson’s disease, e.g., by the treatment with 1-methyl-4-phenyl-1,2,3,6-tetrahydropyridine (MPTP) [108], but patient-derived midbrain cerebral organoids appear to be more informative in studying the pathogenesis of Parkinson’s disease. These midbrain organoids allowed demonstrating the characteristics specific for Parkinson′s disease: impaired differentiation of progenitor cells, reduced number of differentiated dopaminergic neurons, higher number of progenitors, elevated expression of markers of mitophagy and autophagy, appearance of mitochondrial dysfunction, low viability of cells, and dysfunctional response to neuroinflammatory stimuli in LRKK2, DJ-1, or PRKN mutants [105,109,110,111].

**Figure 3:**
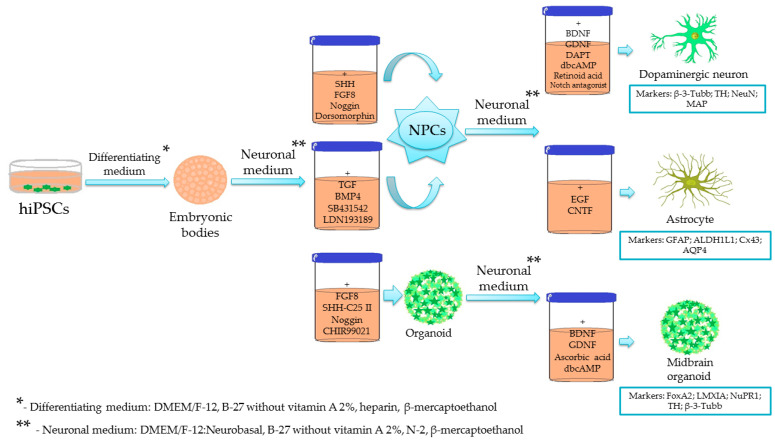
Summary of widely-used protocols for the generation of iPSC-derived midbrain cells and cerebral organoids. The scheme shows the main procedures aimed to establish the appropriate local microenvironment for the in vitro differentiation of cells toward midbrain neurons, glia, and multicellular structures (cerebral organoids).

The general principles of the current methodology used for the generation of iPSC-derived cells and organoids in PD are summarized in Figure 3. As we discussed above, numerous protocols have been applied to get differentiated neuronal and glial cells, or cerebral organoids from human iPSCs. All these protocols have their own strengths and limitations; therefore, the development of novel, probably unified, approaches are a big challenge in modern bioengineering and neurobiology.

Very recently, some new data on the establishment of iPSC-derived brain cells and multicellular ensembles suggest that we might have more efficient tools for deciphering cellular and molecular mechanisms of brain plasticity in (patho)physiological conditions. Some of these revolutionizing protocols are CRISPR-Cas9 generation of iPSC-derived cell lines that are suitable for live imaging and selective isolation of dopaminergic neurons in the culture [112], application of 3D organoids to study the idiopathic form of PD [113], development of in vitro BBB model from iPSCs for the assessment of BBB breakdown in PD [114], generation of brain-on-chips with the microfluidic technologies that are helpful in separating the cell-specific effects or studying the BBB integrity in vitro [115], and establishment of novel cell products matching the requirements for pre-clinical studies or even of clinical-grade quality [116].

## 3. Adult Neurogenesis as a Target for Therapy and Optogenetic Control

Neurogenesis is a well-known target for the pharmaceutical correction of brain plasticity and treatment of neurological and mental disorders [117,118]. Neurogenesis is affected not by drugs or small molecules only [119] but also by other various exogenous stimuli. For instance, restricted sleep results in reduced neurogenesis [120], social interactions promote neurogenesis in the post-ischemic brain [121], and an enriched (multi-stimuli) environment activates neurogenesis in the brain tissue in the postnatal period and leads to obvious effects in NSCs proliferation in physiological aging and Alzheimer’s type neurodegeneration in vivo and in vitro [122,123]. Other factors that affect adult neurogenesis (nutrients, metabolites, hormones, cytokines, etc.) have also been tested as potential modulators of brain plasticity. As an example, lactate produced by niche astrocytes and stem cells, or transported by BMECs from the extra-niche compartment, stimulates adult neurogenesis and mediates the pro-neurogenic effects of physical exercise [124]. Potent regulators of glucose metabolism, such as insulin, insulin-like growth factor-I, glucagon-like peptide-1, and ghrelin, control NSC fate and stimulates SGZ neurogenesis [125]. Pro-inflammatory cytokine IL-6 supports NSCs self-renewal, but a transient surge of systemic IL-6 levels results in an increase in NPCs proliferation and long-lasting depletion of NSCs pools [126]. The stimulation of NAD^+^ synthesis in niche cells leads to the restoration of adult neurogenesis affected in neurodegeneration, presumably due to the activity of NAD^+^-consuming enzymes (NAD^+^-glycohydrolases, poly (ADP-ribose)polymerase) or NAD^+^-dependent sirtuins [127].

Targeting neurogenesis with drugs and compounds affecting some of the above-mentioned regulatory mechanisms is in the focus of neurobiologists and neuropharmacologists. At the same time, given the rapid development of optogenetics, it is not surprising that this method has already been repeatedly tested to modulate the adult neurogenesis with higher precision either in vivo or in vitro [128,129]. Indeed, neural stem or progenitor cells could be transfected with light-sensitive channelrhodopsin2 (ChR2) or other variants of chimeric opsins, for the induction of large photocurrents, either with viral vectors (i.e., lentivirus) or via a non-viral transfection system (i.e., piggyBac transposons). Photostimulation of these cells results in the production of a larger number of neuroblasts and functionally competent neurons in vitro [130], with up-regulated Wnt/β-catenin pathway, or induces differentiation of NPCs into oligodendrocytes and neurons, as well as the polarization of astrocytes to a pro-regenerative/anti-inflammatory phenotype [131]. Expression of ChR2 in human iPSC-derived neuronal cells under the calcium/calmodulin-dependent kinase II (CaMKII) and synapsin 1 (SYN1) promoters was effective in the detection of the differentiated status of the progeny and in the optical control of their growth in vitro [132].

Optogenetic protocols have also been tested in grafted NSCs to increase the expression of genes involved in neurotransmission, neuronal differentiation, axonal guidance, and synaptic plasticity [133]. Cre-lox strategy and piggyBac vectors have been applied for getting the optogenetic stem cell lines from human iPSCs that can switch on optogenetic expression via Cre-induction in vitro for further photomanipulations (activation or silencing) with the differentiated neurons [134]. Embryonic stem cell-derived NPCs stably expressing ChR2 can be efficiently transplanted into the mouse cortex where they show good integration capacity and differentiation toward GABAergic phenotype; however, photostimulation of such optogenetic cells in vivo produce rather controversial effects [135]. In some cases, optogenetics might be used for studying the response of host cells on the transplantation of iPSC-derived neurons: expression of ChR2 in host neurons allows detecting the development of host-to-graft synaptic afferents and establishment of ample output from host cells to the grafted ones [136].

Despite the fact that the role of neurogenesis in the adult brain of humans and non-human primates is still a controversial issue, most neuroscientists believe that the management of neurogenesis could enhance cognitive reserve and stimulate restoration of the brain tissue after injury or in chronic neurodegeneration [10,137,138,139]. Neurobiologists and neurologists are still rather optimistic about using NSCs as a substrate for brain tissue-replacement therapy or stimulating endogenous sources of adult-born neurons for brain tissue repair and facilitation of cognitive functions. As an example, recent experimental data demonstrate that the stimulation of even a small pool of NSCs “rejuvenates” the brain and reduces some age-associated manifestations of cognitive deficits [140], but at the same time, stimulation of neurogenesis may alter forgetting [15].

In PD, impairment of neurogenesis suggests that the effective and long-lasting treatment for PD motor symptoms might be replacing SN dopaminergic cells by means of improved endogenous neurogenesis or by cell-replacement therapy/cell transplantation [40,119]. At present, optogenetic photostimulation has been mainly tested for the modulation of SN neurons. Particularly, light-induced activation of ChR2 dopaminergic neurons in the genetic model Drosophila larva rescues PD symptoms caused by α-synuclein [141], light-dependent activation of mitochondrially expressed proton pump dR reinforces mitochondrial function and prevents α-synuclein-driven mitochondrial dysfunction in a Drosophila model of PD [142]. It should be mentioned that some additional optogenetics-based options became available: light-inducible protein aggregation system that allows photoinduced aggregation of α-synuclein in vitro and in vivo to model the Parkinson’s type neurodegeneration and to find out the effects of abnormal protein accumulation in the brain [143]. Thus, even though there are some positive results of optogenetic manipulations with dopaminergic neurons in experimental PD, there are no examples of a similar approach applied for NSCs/NPCs in PD-neurogenic niches.

## 4. Optogenetic Activation of Niche Astrocytes for the Control of Cell Development in PD

### 4.1. Astrocytes as Potent Regulators of Neurogenesis and Parkinson’s Type Neurodegeneration

The analysis of neurogenesis impairments in neurodegeneration leads to some critical questions: how is it possible to manage the fate of transplanted NSCs/NPCs in the SN since the local microenvironment there supports glial, but not neuronal phenotype acquisition? If so, would the transplantation of mature well-differentiated neurons be the only solution, or could another strategy for precise control of cell proliferation and differentiation within the SN be applied? Taking into consideration the above-mentioned issues, one could propose that for the efficient therapy of PD, two major approaches should be evaluated: (i) stimulation of endogenous neurogenesis in vivo by targeting SN NSCs/NPCs along with the prevention of their development toward the astroglial phenotype; or (ii) obtaining the pool of dopaminergic functionally competent neurons from iPSCs in vitro and their transplantation in SN. Recently, another approach based on the reprogramming of midbrain astrocytes into dopaminergic neurons has been suggested for the treatment of PD [144].

In all cases, astroglial cells could be considered key regulators of neurogenesis and maturation. Glial fibrillary acidic protein (GFAP)-immunopositive radial glial cells (RGCs) located within neurogenic niches are the NSCs that give rise to multipotent and dividing progenitors. In addition, RGS controls cell migration to ensure reparative neurogenesis. The activation of neurogenesis is always associated with an accumulation of astrocytes in neurogenic niches, and the establishment of a niche astroglial network is required for the local microenvironment supporting the proliferation of cell clusters in neurogenic niches [145]. The close contact of niche astrocytes with NSCs/NPCs and brain microvessel endothelial cells (BMECs) of the niche vascular scaffold, as well as secretory activity of astrocytes, control the promotion of neuronal differentiation of stem cells in the SGZ [145,146]. Some data suggest that astrocytes negatively affect neurogenesis and inhibit neuronal differentiation through direct cell-to-cell contacts with NSCs and the modulation of Notch/Jagged1 signaling pathways in an intermediate filament protein GFAP-dependent manner [147].

In PD, midbrain astrocytes play a dual role in the disease progression and tissue repair: (i) astroglial production of reactive oxygen species (ROS) and reactive nitrogen species (RNS) in activated astrocytes partially caused by aberrant expression of α-synuclein (SNCA), parkin (PARK2), protein deglycase DJ-1 (PARK7), and PINK1 genes lead to the damage of dopaminergic neurons and progression of neuroinflammation [148]; (ii) astroglial production of gliotransmitters and neurotrophic factors is important for governing cell proliferation, differentiation, and tissue repair in chronic neurodegeneration [149,150]. Particularly, astrocytes-derived Wnt contributes to dopaminergic neurons survival [151], stimulation of neurogenesis from SN stem cells [152], and tissue regeneration in PD [153] through canonical (Wnt/β-catenin) and non-canonical (Wnt/planar cell polarity and Wnt/Ca^2+^) pathways that are involved in the differentiation of dopaminergic neurons [151].

The activity of astrocyte-derived Wnt is required for NSCs proliferation and differentiation in the SGZ [154], whereas Notch signaling regulates the maintenance of adult NSCs governing them out of cell cycle exit, thereby decreasing the pool of NPCs [155]. Thus, Notch signaling prevents excessive recruiting of NSCs, while Wnt signaling supports the proliferation and differentiation of NPCs and neuroblasts. Recent data reveal novel aspects of Notch and Wnt signaling in NSCs development: when iPSCs cortical spheroids are treated with Wnt and Notch modulators, they demonstrate a synergistic effect on neural regional patterning and occurrence of neurogenesis and gliogenesis (increase in Notch and Wnt activity results in the development of a larger number of glial cells), thus, repressing impact of Notch inhibitor on Wnt inhibition and the positive impact of Wnt activation on Notch signaling are proposed [156]. It should be noted that Wnt signaling is a well-known target for the activity of proteins involved in the pathogenesis of PD: in the healthy brain, LRKK2 (product of *PARK8* gene) serves as a scaffold protein and positive regulator of the canonical pathways, whereas parkin (product of PARK2 gene) induces β-catenin degradation and suppression of the canonical pathway [157]. Thus, in physiological conditions, parkin protects dopaminergic neurons from excessive activation of the Wnt/β-catenin pathway [158], but in PD, this mechanism is lost due to parkin alterations. Thus, the data on enhanced neurogenesis due to Wnt/β-catenin overactivation associated with impaired differentiation of dopaminergic neurons [159] are rather reasonable. The stimulatory effect of LRRK2 on the non-canonical Wnt/planar cell polarity pathway was reported as well [160].

Other than Wnt/β-catenin signaling, FGF8 plays a great role in the regulation of differentiation toward dopaminergic phenotype: FGF receptors (FGFRs) regulate the self-renewal and dopaminergic differentiation of NPCs in the developing midbrain [161,162,163]. Dysfunction of the FGF-driven mechanisms of midbrain development and control of midbrain neurons survival and metabolism is implicated in the pathogenesis of PD [163]. It was reported that the dopaminergic differentiation of embryonic stem cells in vitro could be facilitated by astrocytes providing FGF. Moreover, the optogenetic activation of astrocytes transplanted in SN in vivo results in elevated FGF release and promotion of appropriate differentiation of co-transplanted stem cells [161]. Novel optogenetics tools, such as optoFGFR based on the cryptochrome2 domain and cytoplasmic region of FGFR, enable light-guided activation and clustering of FGFRs for efficient analyzing of the downstream molecular events [164,165]. We suggest that a similar approach could be tested to modulate the FGF-driven regulation of stem cell development and dopaminergic differentiation in PD.

### 4.2. Optogenetic Targeting of GFAP^+^ Cells in the Neurogenic Niche: Established and Prospective Approaches to Cells Activation and Signal Propagation

The essence of astroglial activation is the elevation of intracellular Ca^2+^ levels due to Ca^2+^ influx through membrane channels, i.e., L-type voltage-operated calcium channels, VOCC [166], connexin 43 (Cx43) hemichannels [167], and transient receptor potential channels, TRP [168], or Ca^2+^ release from intracellular stores (endoplasmic reticulum, mitochondria, nucleus) via activation of inositol-3-phosphate receptors of cyclic ADP-ribose-sensitive ryanodine receptors [169,170]. The activation of Ca^2+^ release mechanisms is a result of stimulation of astroglial Gq, Gi/o, or Gs G-protein-coupled receptors (GPCRs) culminating in the synthesis of second messengers with Ca^2+^-mobilizing activity, whereas the opening of VOCC is triggered by high extracellular concentrations of glutamate, K^+^, and ATP, i.e., in active brain regions or in inflammatory loci [166,171]. Thus, “artificial” induction of Ca^2+^ rise in astroglial cells might mimic the activation achieved by ligands of GPCRs, K^+^, ATP, or cytokines. As a result of activation, extracellular K+ concentrations transiently rise, astrocytes release gliotransmitters and change their mitochondrial activity and proliferative status [172,173,174]. Actually, this is a principle of optogenetic photostimulation of astroglial cells expressing ChR2 or optoGPCRs under the astroglial promoters (i.e., GFAP), which recently appeared as a new approach to control brain activity [175,176,177,178].

Midbrain astrocytes in PD with SNCA mutations demonstrate aberrant Ca^2+^ release from intracellular stores into cytosol, presumably, caused by mitochondrial dysfunction [90]. Thus, one may propose that optogenetic stimulation of PD-specific astrocytes with the mutant form of SNCA would result in an abnormal pattern of their activation.

It should be kept in mind that recent complex proteomic and transcriptomic analyses revealed interesting differences in the expression pattern of astrocytes in various brain regions. Particularly, hippocampal astrocytes and striatal astrocytes predominantly express GFAP or µ-crystalline, respectively, and they are different in gap-junctional coupling (lower in striatal astroglia) and GPCR-mediated Ca^2+^ signals (weaker response in hippocampal astrocytes) [179]. Thus, any, including optogenetic, manipulations with hippocampal and striatal astroglial cells would have a priori different efficacy and results: expression of light-sensitive molecules under the GFAP promoter would be higher in the hippocampus, but Ca^2+^-driven activation of glial cells would be more evident in the striatum.

The expression of light-sensitive molecules under the control of the astroglial promoter (GFAP) raises the question of what type of cells within the neurogenic niche could be affected by photostimulation. Even though there is a heterogeneity of astroglial cells, the expression of GFAP could be easily detected in the majority of mature resting and reactive astrocytes throughout the brain [180]. Higher expression of GFAP in reactive astrocytes, particularly in neurodegeneration [181], makes it possible to increase the efficacy of photostimulation in the affected brain vs. the healthy brain. Indeed, optogenetic protocols targeting astrocytes provide precise manipulation with their functional status, secretory phenotype, and interactions with mature neurons in physiological conditions and in neurodegeneration [175,177,182,183].

Less is known about the application of optogenetics for controlling astroglia-driven regulation of adult neurogenesis. We have demonstrated before that optogenetic stimulation of niche astrocytes expressing channelrhodopsin-2 under the GFAP promoter was efficient in activating the neurogenic potential of NSCs/NPCs in the in vitro neurogenic niche model or in implanted intrahippocampal neurospheres ex vivo in experimental Alzheimer’s disease [184,185]. Photostimulation of iPSC-derived ChR2-expressing astrocytes co-cultured with iPSC-derived neurons results in effective bidirectional signaling, which is important for supporting the maturation of neurons and the establishment of a functional synaptic network, even though the transcriptomic analysis confirms that iPSCs-originated astrocytes are relatively immature compared to adult cortical astrocytes [186].

Quiescent NSCs, as RGs, demonstrates the expression pattern as GFAP^+^Nestin^+^PCNA^−^Pax6^+^NeuroD1^−^. Type-1 NSCs, as slowly dividing, cells have the phenotype GFAP^+/−^Nestin^+^PCNA^+^Pax6^+^NeuroD1^−^ and express lower GFAP. NPCs, as amplifying progenitors, with the phenotype GFAP^−^Nestin^+^PCNA^+^Pax6^+^NeuroD1^+^ do not express GFAP during neurogenesis [187]. Thus, the expression of light-activated molecules in NSCs under the GFAP promoter could regulate their activity. However, it might be impossible to use the same strategy to express a construct in post-mitotic astrocytes and NSCs: adeno-associated viruses (AAV) used as vectors are inefficient in transducing stem cells; therefore, engineered AAV variants or other delivery tools (i.e., polymer complexes containing plasmids, episomes, or retrovirus- and lentivirus-based vectors) should be applied [188,189,190,191].

The most attractive feature of optogenetic protocols is an opportunity to stimulate the particular cell precisely and in a controllable manner. While considering astroglial optogenetic stimulation, one should remember the existence of the so-called astroglial syncytium due to the activity of intercellular gap junctions [192]. It is well-known that Ca^2+^ waves in astrocytes propagate via gap junctions consisted of connexin 43 (Cx43) channels [193], thereby resulting in the activation of astroglia located distantly from the focus of primary activation [194] or via extracellular ATP-dependent mechanisms [193]. However, whether or not this mechanism is relevant in optogenetically-stimulated astrocytes remains to be evaluated. The photostimulation of astrocytes expressing light-gated glutamate receptor 6 (LiGluR) in vitro results in the activation of adjacent non-expressing LiGluR astrocytes; this effect was insensitive to the blockers of gap junctions but sensitive to inhibitors of ATP-driven purinergic signaling [195]. Taking into consideration that reactive astrocytes have permissive conditions for Ca^2+^-dependent ATP release [196], one could suggest that in a neurodegeneration-affected brain, the propagation of light-induced signals from the particular astrocytes would be facilitated.

In a rat rotenone-induced model of PD, increased expression of Cx43 was detected in SN, striatum, and basal ganglia astrocytes [197], thus suggesting that metabolic and functional coupling of astroglial cells might be enhanced in Parkinson’s type neurodegeneration. The same phenomenon is evident in brain ischemia [198] and Alzheimer’s disease [199] and might reflect the neuroprotective potential of reactive astrocytes, or could be a consequence of Cx43 functional coupling with another protein, CD38/NAD^+^-glycohydrolase [200], whose expression is elevated in neuroinflammation and neurodegeneration [199]. This suggestion turns us to the idea of NAD^+^-dependent mechanisms in the molecular pathogenesis of PD [201]. Since Cx43 may act as an NAD^+^-transporting molecule in the plasma membrane [202], higher expression of Cx43 might be beneficial for cell survival. Probably, this is a reason why NSCs/NPCs express up-regulated functional Cx43 in brain injury [203]. It is interesting that when NSCs are engrafted into the striatum, they express much higher levels of Cx43, and host cells do the same within the limited period of time [204]; thus, it could be utilized for the improvement of transplantation outcomes. Indeed, the positive effect of gap junction-mediated communication between host cells and NSCs was demonstrated in organotypic slice cultures [205]. Along with this idea, the optogenetic control of cell engraftment might be rather useful: photostimulation of transplanted neurons helps in the assessment of their functional integration into neuronal circuits and communication with other types of cells in the host brain tissue [206]. However, such an approach has never been tested for astroglial cells.

The elevated expression of Cx43 might not correspond to facilitated intercellular communication only: membrane Cx43 hemichannels serve as efflux-oriented transporters for NAD^+^, lactate, or Ca^2+^ in astrocytes; therefore, they provide release of low molecular weight substances and ions into extracellular space with no apparent effect on direct cell-to-cell communication [207]. The activity of this machinery is abnormal in PD: α-synuclein induces the opening of Cx43 hemichannels, excessive Ca2+ rise in the cytosol, gliotransmitters, and cytokines release [208]. In sum, astroglial cells in PD might respond to photostimulation-induced Ca^2+^ intracellular elevations to a greater extent than normal cells due to higher expression of gap junction proteins of Cx43 hemichannels. What might be an outcome for such effects within the neurogenic niche or in the affected brain regions remains to be evaluated. However, experimental data on elevated expression and activity of Cx43 hemichannels driving better communication of host cells and transplanted NSCs [209] allow considering Cx43 hemichannels as a target for light-guided control of engrafting efficacy. Since the normalization of neuron-astroglial gap junction-mediated crosstalk by optogenetic manipulations with astrocytes was proposed in [210], a similar approach should be tested for niche astrocytes communicating with stem cell grafts.

We have proposed before [211] that Cx43 expression in different cells of the neurogenic niche (radial glia, mature astrocytes, endothelial cells) could be utilized to control neurogenesis. Indeed, the expression of Cx43 is indispensable for RGs proliferation in the adult hippocampus [212]. Neuroectodermal specialization of embryonic stem cells depends on the rate of Cx43 expression [213]. Deletion of Cx43 suppresses hippocampal adult neurogenesis due to the inhibition of NSCs proliferation and survival [212], whereas the absence of Cx43 expression in NPCs results in their predominant differentiation toward a neuronal, but not astroglial, phenotype, probably, due to increased β-catenin signaling and Wnt-driven expression of pro-neuronal genes [214]. Indeed, Cx43 in NPCs down-regulates β-catenin signaling, reduces the proliferation of progenitors, and promotes astroglial differentiation [215].

It should be noted that in resting astrocytes, Cx43 localizes in intracellular vesicles, but the activation of cells drives Cx43 expression at the plasma membrane contact sites [216]. Cx43 interacts with β-catenin directly, and the activation of Wnt signaling results in the re-dislocation of Cx43 in some cell lines leading to enhanced expression of Cx43 in the nucleus, but not at the plasma membrane or cytosol [217]. The same phenomenon has not been reproduced in NSCs/NPCs yet, but it might be tempting to speculate that the positive effects of Wnt on dopaminergic neuron generation and survival are disrupted in PD due to the abnormal activity of parkin and the overactivity of the Wnt/β-catenin signaling pathway could be modulated via Cx43-β-catenin interactions at the plasma membrane of NSCs/NPCs.

Since neuronal activity increases Cx43 expression in astrocytes [218], and excitatory (NMDA, or depolarizing action of GABA) stimuli directly promote differentiation of NPCs toward neuronal phenotypes [6,219], it is tempting to speculate that the establishment of an in vitro neurogenic niche model with mature neurons or with conditions mimicking excitation/inhibition balance specific for neurogenic niches, would give us new opportunities in increasing the efficacy of astroglial (photo)activation for the local control of neurogenesis.

Application of up-to-date protocols for optical mapping of gap junctions, for instance, PARIS, “pairing actuators and receivers to optically isolate gap junctions” [220] or optoGap, an optogenetics-based tool for the analysis of cell-to-cell connexin-driven coupling [221], would be helpful in further elucidating the Cx43 activity in NSCs/NPCs and niche astrocytes.

## 5. Alternative Approaches to Restoring Impaired Neurogenesis in PD

In the context of PD pathogenesis, motor dysfunction appears as a result of excessive GABAergic output in the striatum: normally, dopaminergic neurons of SN terminate at the striatum and release dopamine there to propagate signals to cholinergic and GABAergic neurons, resulting in the inhibition of the output from GABAergic neurons [222,223]. In the healthy striatum, the majority of cells are the GABAergic interneurons, whereas the role of striatal dopaminergic cells is not clear [224].

New dopaminergic neurons adjacent to the band of preserved nigral input and expressing tyrosine hydroxylase and dopamine transporter have been found in the striatum of PD patients [54,225]. Optogenetic activation of striatal tyrosine hydroxylase-expressing interneurons in mice in vivo produce strong GABAergic inhibition, but no evidence for dopamine production has been obtained [226]. Loss of nigrostriatal innervation results in morphological and functional changes in this population of cells aimed to compensate the GABAergic inhibition [227]. Probably, endogenous dopamine negatively controls the number of these cells [228]. Currently, optogenetic activation of striatal neurons was found to be an efficient tool for studying striatum-dependent neurological processes, i.e., reward behavioral encoding, reinforcement learning, and motivation, as it was reviewed in detail elsewhere [229].

Insulin receptors are expressed on midbrain dopamine neurons, so their stimulation controls dopaminergic transmission in the striatum [230]. Particularly, insulin may enhance dopamine release in the striatum through cholinergic interneurons [231]. Data obtained in Drosophila reveal that the induction of NSCs from glia, their proliferation and limited neurogenesis are regulated by insulin signaling [232]. Neurogenesis in conventional neurogenic niches (SVZ and SGZ) also depends on insulin and insulin-like growth factors (IGF) [233]; thus, cerebral insulin resistance evident in chronic neurodegeneration (Alzheimer’s disease, Parkinson’s disease) negatively affects neurogenesis [234], whereas peptides facilitating insulin effects promote the development of new dopaminergic neurons in SN in a model of PD [235]. Thus, the modulation of insulin signaling in SN and striatum might be important for restoring neurogenesis in these non-conventional neurogenic niches. In this context, the photodynamic reversible opening of the blood-brain barrier (BBB) [236] might be useful for driving insulin or insulin-like growth factors transport into the particular brain region, as it was shown for IGF-I in the active brain [237].

Neuroblasts differentiating into mature GABAergic interneurons have been found in the striatum close to the SVZ in the adult brain in humans, and local neurogenic events here are diminished in Huntington’s disease [238]. Since dopaminergic activity is required for stimulating the striatal neurogenesis [65], the loss of SN neurons in PD would result in the suppression of striatal neurogenesis. However, alternative hypotheses on the origin of adult-born striatal interneurons have been proposed, including differentiation of local NPCs [239,240] or conversion of striatal astrocytes into mature neurons by blocking Notch signaling [241].

The latter approach has attracted a lot of attention in recent years because the direct reprogramming of adult post-mitotic cells might be quite useful in the replacement of lost neurons with new cells in brain regions (i.e., SN and striatum) with very limited neurogenic capacity in adults [242]. However, the conversion of SN GFAP^+^ cells into neurons was found, thereby providing an alternative neurogenic mechanism within SN and striatum: GFAP^+^/s100β^+^ astrocytes could transdifferentiate into dividing cells (neuroblasts) or even dedifferentiate back to NSCs [64].

In the adult mouse brain, transcription factor SOX2 can induce the transformation of astrocytes into neuroblasts that can be further driven to mature neurons with BDNF or valproic acid as a histone deacetylase inhibitor [243]. The induction of expression of Ascl1 in cortex astrocytes results in the formation of GABAergic neurons, while Neurog2 expression is responsible for the glutamatergic phenotype, but NeuroD4 is capable of reprogramming astrocytes into neurons that cannot complete synaptic maturation [244]. The combination of several transcription factors, NEUROD1, ASCL1, and LMX1A, and the microRNA miR218 is helpful in the in vivo and in vitro reprogramming of striatal astrocytes of mouse and human origin into functional dopaminergic neurons [245]. Moreover, when small molecules that promote chromatin remodeling and activate the TGFβ, Shh, and Wnt signaling pathways, such as ascorbic acid, valproic acid, or 5-aza-2′-deoxycytidine, TGF and BMP4 inhibitors SB431542 and LDN193189, sonic hedgehog (SHH) and the GSK3β inhibitor CT99021, dual-Smad inhibitors SB431542 and LDN193189, and midbrain patterning signals CT99021 and purmorphamine, have been applied, the number of tyrosine hydroxylase-expressing neurons was increased [245]. It is important to note that the in vivo reprogramming of striatal astrocytes into dopaminergic neurons results in the improvement of behavioral characteristics of 6-OHDA-treated mice with PD [245]. The reconstruction of nigrostriatal circuits, replenishment of dopaminergic neurons and reduction of neurological deficits were achieved in mice with a 6-OHDA model of PD by the reprogramming of astrocytes to functional neurons via depletion of RNA-binding protein PTB (PTBP1), and the functional characteristics of newly-developed neurons were confirmed with the chemogenetic protocols [144]. Astrocytes of different origins might demonstrate various abilities to be reprogrammed into neurons: adult human astrocytes could be reprogrammed to neuroblasts with miRNAs (miR-302/367), but mouse astrocytes required valproic acid for successful conversion [246].

When mouse embryonic bodies (EBs) were transplanted into SN of rats and mice, stimulation of neurogenesis was observed, but the establishment of fully differentiated dopaminergic neurons failed; however, previously non-dividing resident GFAP+/S100b+ cells acquired neuroblast markers after EBs transplantation [64]. Similarly, in the 6-OHDA rat model of PD, chronic (10 days) infusion of platelet-derived growth factor (PDGF-BB) and brain-derived neurotrophic factor (BDNF) results in the generation of newly-formed cells in the striatum and SN, but these cells do not demonstrate the expression pattern of striatal mature projection neurons or dopaminergic neurons in SN [247]. The combination of 6-OHDA-lesion of SN dopaminergic neurons and infusions of transforming growth factor α (TGFα) into forebrain structures results in a massive migration of neural progenitors from the SVZ to the striatum, their differentiation to dopaminergic neurons, and the improvement of rotational behavior in rats [248]. However, data obtained in humans and in rodents with PD models seem to be controversial: the striatum of PD patients was found to contain six times fewer tyrosine hydroxylase-expressing cells [249]. Moreover, as it was resumed in [250], there is no confirmation that the enhancement of striatal neurogenesis would result in the improvement of behavioral effects in PD in a similar way, as it was shown in the striatal transplantation of dopaminergic neurons, but the stimulation of striatal neurogenesis and reinnervation of local interneurons is a promising strategy in PD.

Attempts to produce dopaminergic neurons from SVZ stem cells have shown that adult NSC-derived cells co-express Nestin and tyrosine hydroxylase and demonstrated a low survival rate, but embryonic stem cell-derived neurons have characteristics of mature cells with strong dopamine release upon the action of depolarizing stimuli [251]. Thus, the use of stem cells close to embryonic parameters (iPSCs) should have obvious advantages in cell-replacement therapy.

Undoubtedly, all the attempts aimed to reduce α-synuclein-induced alterations (including prevention of its aggregation and dissemination or enhancing degradation of α-synuclein aggregates) would be efficient in restoring the neurogenesis in PD-affected brains, thereby contributing to functional recovery [29].

## 6. Conclusion and Perspectives

The current attempts to establish reliable and safe therapeutic platforms for the restoration of impaired brain plasticity in neurodegeneration are facing the complexity of adult neurogenesis. Recent achievements in understanding the key molecular mechanisms of NSCs/NPCs maintenance and development, the role of intercellular communications in the adjustment of neurogenesis to the actual needs of the active brain, and application of up-to-date tools for getting the desired cellular phenotypes (e.g., in iPSC-based protocols) and precise activation of target cells (e.g., in optogenetic protocols) suggest new opportunities in the cell-replacement therapy, either via the stimulation of endogenous neurogenesis or the generation of cells for efficient engrafting.

In the case of synucleinopathies, this approach should be based on the molecular mechanisms of impaired brain plasticity caused by abnormal accumulation and distribution of α-synuclein in various brain regions, including conventional and non-conventional neurogenic niches. There are no doubts that aberrant neurogenesis is ultimately involved in the pathogenesis of PD from the very early, even pre-motor and pre-manifesting, stages. Correct analysis of neurogenesis impairments, as well as the development of novel approaches to manipulate the neurogenic capacity of NSCs/NPCs, would give progress in the early diagnostics, effective prevention, and treatment of PD.

In this context, various niche cellular components (NSCs, NPCs, astrocytes, BMECs, and mature neurons) serve as promising targets for the optogenetic control of the local microenvironment. Modulating the functional activity of niche cells might be helpful in the control of cell proliferation, reprogramming, and differentiation either in vitro or in vivo. The same approach is rather prospective for improving the outcomes of cells transplantation and their functional integration in the affected brain regions.

Thus, the application of novel optogenetic/chemogenetic tools and advanced in vitro models, including those based on iPSC-derived cells, organoids, or utilizing 3D brain-on-chip platforms, are of great importance for the development of new therapeutic options and assessment of aberrant neurogenesis in Parkinson’s type neurodegeneration.

## Abbreviations

AV	adeno-associated virus
ALDH1L1	aldehyde dehydrogenase 1 family member L1
AMP	adenosine monophosphate
ADP	adenosine diphosphate
AQ4	aquaporin 4
ASCL1	Achaete-Scute homolog 1
ATP	adenosine triphosphate
BBB	blood-brain barrier
BDNF	brain-derived neurotrophic factor
BMECs	brain microvessel endothelial cells
BMP4	bone morphogenetic protein 4
CaMKII	calcium/calmodulin-dependent kinase II
CD	cluster of differentiation
ChR2	channelrhodopsin 2
CNTF	ciliary neurotrophic factor
CRISPR-Cas9	clustered regularly interspaced short palindromic repeats-associated protein 9
Cx	connexin
3D	three-dimensional
DAMPs	damage-associated molecular patterns
DJ-1	deglycase-1
EB	embryonic body
EGF	epidermal growth factor
E/I	excitation/inhibition balance
FBS	fetal bovine serum
FCS	fetal calf serum
FGF	fibroblast growth factor
Foxa2	forkhead box protein A2
GABA	gamma-aminobutyric acid
GDNF	glia cell line-derived neurotrophic factor
GFAP	glial fibrillary acidic protein
GPCRs	G-protein-coupled receptors
GSK3	glycogen synthase kinase 3
HDAC	histone deacetylase
IGF	insulin-like growth factor
IL	interleukin
iPSCs	induced pluripotent stem cells
LIF	leukemia inhibitory factor
LiGluR	light-gated glutamate receptor 6
Lmx1a	LIM homeobox transcription factor 1 alpha
LRKK2	leucine-rich repeat kinase 2
MPTP	1-methyl-4-phenyl-1,2,3,6-tetrahydropyridine
mRNA	messenger ribonucleic acid
NAD^+^	nicotinamide adenine dinucleotide
NeuroD1	neurogenic differentiation 1 transcription factor
Neurog2	neurogenin 2
NFIA	nuclear factor I A
NFIB	nuclear factor I B
NMDA	N-methyl-D-aspartate
NPCs	neural progenitor cells
NSCs	neural stem cells
Nurr1	nuclear receptor related 1
6-OHDA	6-hydroxydopamine
optoFGFR	light-activatable fibroblast growth factor receptor
optoGap	light-activatable gap junction
optoGPCR	light-activatable G-protein coupled receptor
PARK2	parkin ubiquitin protein ligase
Pax6	transcription factor paired box protein
PCNA	proliferating cell nuclear antigen
PD	Parkinson’s disease
PDGF-BB	platelet-derived growth factor
PINK-1	PTEN (phosphatase and tensin homolog deleted)-induced kinase 1
PRKN	parkin
RGCs	radial glia cells
PTBP1	RNA-binding protein PTB
RNS	reactive nitrogen species
ROS	reactive oxygen species
S100β	S100 calcium-binding protein B
SGZ	subgranular zone
SHH	sonic hedgehog
smNPCs	small molecule-treated neural progenitor cells
SN	substantia nigra
SNCA	synuclein
Sox6	SRY-Box transcription factor 6
Sox9	SRY-Box transcription factor 9
SVZ	subventricular zone
SYN1	synapsin 1
TGFβ	transforming growth factor-beta
TRP	transient receptor potential channel
VOCC	voltage-operated calcium channel
Wnt	Wingless/Int-1
